# Processing and Mechanical Properties of Macro Polyamide Fiber Reinforced Concrete

**DOI:** 10.3390/ma7127634

**Published:** 2014-11-26

**Authors:** Joong Kyu Jeon, WooSeok Kim, Chan Ki Jeon, Jin Cheol Kim

**Affiliations:** 1 Research and Business Development Center, Kolon Global Corp, Pogok-eup, Cheoin-gu, Yongin-si, Gyeonggi-do 449-815, Korea; E-Mail: jkjeon31@kolon.comd; 2 Department of Civil Engineering, Chungnam National University, 99, Daehak-ro, Yuseong-gu, Daejeon 305-764, Korea; 3 Department of Urban Construction Engineering, Incheon National University, 119, Academy-ro, Yeonsu-gu, Incheon 406-772, Korea; E-Mail: johnland@incheon.ac.kr; 4 Expressway and Transportation Research Institute, Korea Expressway Corp. 208-96, Dongbu-daero 922 beon-gil, Dongtan-myeon, Hwaseong-si, Gyeonggi-do 445-810, Korea; E-Mail: jckim@ex.co.kr

**Keywords:** fiber, polyamide, mechanical properties, mechanical testing, extrusion

## Abstract

This study developed a macro-sized polyamide (PA) fiber for concrete reinforcement and investigated the influence of the PA fiber on flexural responses in accordance with ASTM standards. PA fibers are advantageous compared to steel fibers that are corrosive and gravitated. The macro-sized PA fiber significantly improved concrete ductility and toughness. Unlike steel fibers, the PA fibers produced two peak bending strengths. The first-peaks occurred near 0.005 mm of deflection and decreased up to 0.5 mm of deflection. Then the bending strength increased up to second-peaks until the deflections reached between 1.0 and 1.5 mm. The averaged flexural responses revealed that PA fiber content did not significantly influence flexural responses before *L*/600, but had significant influence thereafter. Toughness performance levels were also determined, and the results indicated more than Level II at *L*/600 and Level IV at others.

## 1. Introduction

Randomly distributed short fiber reinforcements are used to improve the brittle characteristics of concrete. The reinforcements, including steel fibers [1,2,3,4,5], carbon fibers and polymer fibers [6,7,8,9,10], are used to control the initiation and propagation of cracks [11,12,13,14,15]. The fibers hold together the cracks and reduce potential problems such as water permeating causing steel corrosion and deterioration. The resulting fiber reinforced concrete (FRC) exhibits superior performance compared to plain concrete due to its high tensile strength and ductile tensile behavior. Application of FRCs is increasing recently in areas requiring extreme mechanical and environmental loadings.

Steel fiber reinforced concrete has both high tensile strength and bending strength and controls cracks. However, gravitation and corrosion of the steel fiber may occur, and rebounding during tunnel shotcrete application due to low adhesion characteristics causes lower than expected strength and higher costs [16]. Organic fiber usually has lower elastic modulus and tensile strength compared to steel fiber and is easily tangled, causing low workability. Thus, for practical use in reinforced concrete, organic fiber requires improved mechanical characteristics and workability.

This study developed polyamide fibers with optimized features for reinforcing concrete, to improve the mechanical properties and workability of organic fiber reinforced concrete. Typically, concrete is comprised of multiple phases, including micron-scale phases for the C-S-H gel, millimeter-scale for sands, and centimeter-scale for aggregates, and this multiphase composition results in complex-cracking behavior [17,18]. However, since cracks usually occur along the aggregates, it is consequently necessary for reinforcing fiber to perform in the macro-scale. Thus, this study developed and investigated macro-sized, or bundled, polyamide (PA) fiber, tested and optimized the PA fiber bundles, and investigated the properties of PA fiber reinforced concrete (PAFRC).

The polyamide fiber bundles were made of approximately 384 micro-size polyamide fibers (diameter = 19.5 μm), and the bundled format improved adhesion to cement paste. Polyamide fiber has improved mechanical properties compared to polypropylene (PP) fiber, and lower weight and density and no corrosion compared to steel fiber. Polypropylene fiber also gets popularity due to concrete performance improvement under crack opening and slippage [4]. These improved mechanical properties for compression, tension, flexure, impact blows and plastic shrinkage cracking [5] and adhesion characteristics lead to improved workability and less rebounding when spouting shotcrete.

The objective of this study is to provide the PA fiber processing technique and to report the physical and mechanical properties of PAFRC in terms of compressive and flexural responses in accordance with ASTM C1609/C 1609M-05 and C1018-97 [19,20].

## 2. Materials and Methods

### 2.1. Macro PA Fiber Development and Processing

**Figure 1:**
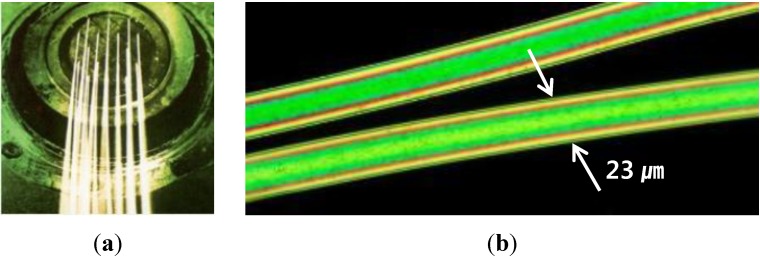
Picture of micro polyamide fibers. (**a**) Single polyamide fiber production; (**b**) magnification of single polyamide fiber.

**Figure 2:**
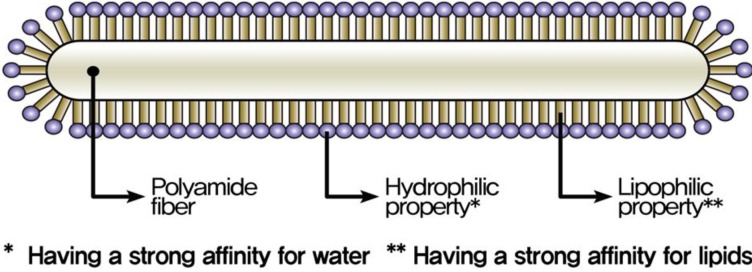
PA fiber (Diameter: 23 μm) with dispersant coating.

Polyamide (PA) fiber is one of the synthetic fibers commonly used in textiles. A micro-size polyamide fiber is presented in Figure 1. Polyamide fibers are produced through a shaped nozzle, and the thickness of the fiber is constant throughout its length. A dispersant is used during the production of PA filaments to prevent the fibers tangling, as shown in Figure 2. The dispersant is composed of hydrophilic and lipophilic groups made of 40% to 50% polyalcohol ester lubricant, 30% to 40% nonionic surfactant solution and 10% to 30% anti-static agent. In concrete, the hydrophilic group, having a negative charge, causes repulsion between fibers, prevents the fibers tangling, and improves fiber dispersion. The hydrophilic group induces hydrogen bonding and leads to improved adhesion between the fibers and cement paste. Also, the dispersant coating during the filament production decreases fiber elongation and increases fiber strength.

**Figure 3:**
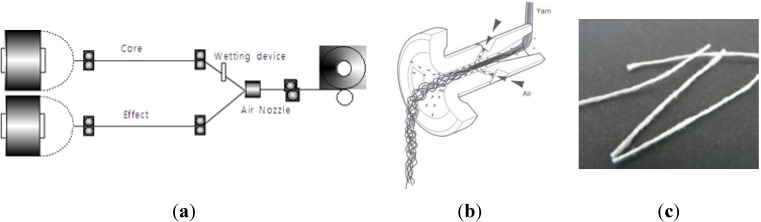
Production process of bundled PA fiber using air textured yarn. (**a**) Air texturing process; (**b**) air nozzle; (**c**) PA fiber (30 mm × 0.47 mm).

**Figure 4:**
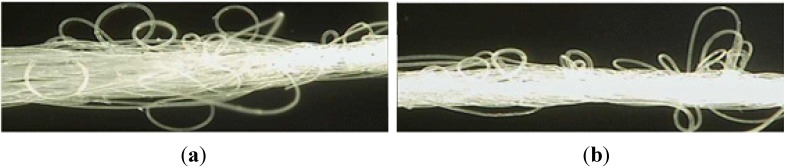
PA fiber processed by air textured yarn (Optical microscope ×8). (**a**) PA fiber before temperature treatment; (**b**) PA fiber after temperature treatment.

This study used a fiber fabrication method of air-textured yarn to develop bundled PA fibers in a macro size. In the production process two-polyamide filaments, one for the core yarn and one for the effect yarn (see Figure 3a), are injected into the nozzle with oblique high-pressure air-flow (see Figure 3b). The injection speed of the effect yarn is slower than the core yarn and a number of loops are subsequently formed due to the difference in injection air-flow speed. After passing the air nozzle, the effect yarn length is longer than the core yarn, and the fiber is then stabilized with a proper temperature treatment, preventing separation of the yarn. Polyamide fiber produced by the air textured yarn method is shown in Figure 4. The loops and bulking of the textured yarn allows cement paste to pass through the yarn, and produces an increased surface area for contact between the cement paste and yarns, resulting in better adhesion. It is expected that this improved adhesion property will produce higher flexural responses compared to polypropylene (PP) fibers, which are mono-filaments.

The bundled PA fibers developed by this study have a larger fiber size and greater number of filaments compared to PP fibers and other organic fibers. To determine the number of PA filaments needed to fit the desired mechanical properties, this study tested and optimized the number of PA filaments at 1,800 Denier and 384 filaments of PA fiber. The mechanical properties of the PA fiber are tabulated in Table 1 and compared to polypropylene (PP) fibers. For PP fibers, BarChip Macro produced by Elasto Plastic Concrete [21], which is a synthetic fiber frequently used in Europe [18], was selected.

**Table 1:** Material properties of PA and PP fibers.

Fiber Type	Density (g/cm^3^)	Length (mm)	Diameter (mm)	Elastic Modulus (GPa)	Tensile Strength (MPa)
PA	1.14	30	0.47	Min. 3	650
*PP	0.9–0.92	42	**Rec.	8.2	550

* PP: BarChip Macro fiber; ** Rec.: rectangular section of 1.0 mm × 0.5 mm.

### 2.2. Experimental Program for Pull-Out Test

**Figure 5:**
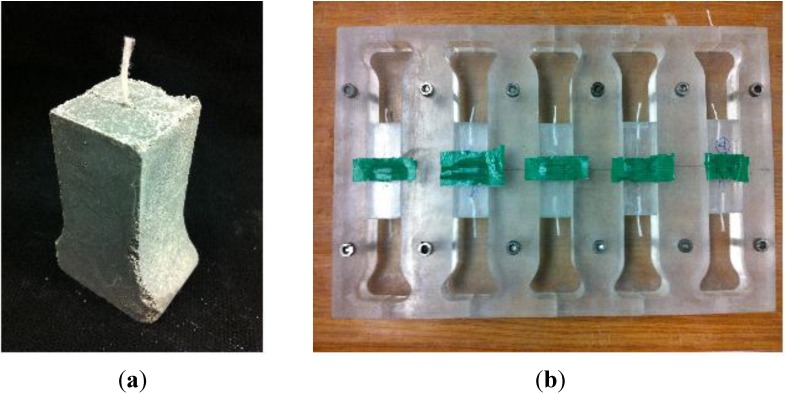
Pictures of pull-out test specimen and mold. (**a**) Pull-out test specimen; (**b**) pull-out test mold.

A pull-out test of a macro-size PA fiber was conducted to determine the fiber-to-matrix bond behavior, as shown in Figure 5. The bundled PA fibers have a larger specific surface area, which accommodates attachment to cement, compared to PP fibers and other fiber reinforcements. The PA fibers used in the test had Ф0.47 mm and the length of the fiber embedded in the matrix (25 × 25 mm) was 15 mm (half of the total PA fiber length). A total of four specimens were prepared for the test. Similar to the method used in previous research [22], the pull-out load was directly measured from the load cell in the cross head, and the displacement or slip of the PA fiber was measured using a linear variable differential transformer (LVDT).

The weight ratios of cement matrix used in the pull-out test were 0.628, 1.0, 0.25, 2.3, 0.00089 and 0.0079 for water, ordinary portland cement (OPC), fly ash, sand, air-entraining agents (AEA) and water-reducing admixture (WRA). The compressive strength of the specimen on the 28th day was 58.09 MPa. In the cement matrix, only fine aggregates with AEA were used with OPC. An appropriate amount of WRA was used to meet the target slump of 120 ± 25 mm, which is standard for tunnel shotcrete. The mixture was mixed with PA fibers and placed in a mold for three days. After demolding, the specimens were cured at room temperature. All specimens were tested on the 28th day.

### 2.3. Experimental Program for Flexural Bending Test

**Figure 6:**
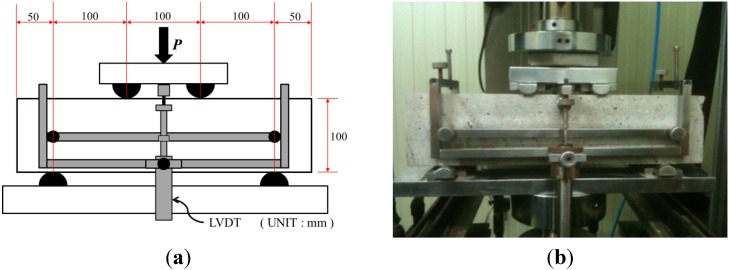
Bending specimen geometry and test set-up. (**a**) Specimen geometry; (**b**) Test set-up picture.

The schematic and picture of the flexural test set-up are presented in Figure 6. An INSTRON 5582 testing machine (DatapointLabs, Ithaca, NY, USA) was used in a laboratory for this flexural test. The rate of deflection was controlled to 0.1 mm/min after *L/*900 (=0.333 mm) with the sampling rate of 16 Hz (≥2.5 Hz). The boundary conditions follow ASTM Testing Method C78 [23], which allows supporting rollers rotating on their axes. The beam specimen size of 100 × 100 × 400 mm with a span length (*L*) of 300 mm was determined in accordance with ASTM C1609/C 1609M-05 [20]. The specimen was built with dimensions of 150 × 150 × 550 mm and then cut into the size 100 × 100 × 400 mm (*L* = 300 mm) to prevent the fiber aligning parallel to the direction of the beam length, and to place the fiber randomly. A steel frame was attached at the neutral axis of the beam specimen to measure the net deflection of the beam using two LVDTs at both sides. (Only one LVDT is shown in Figure 6 and the other one is located at the other side of the beam.) Applied load (P) was measured directly from the load cell in the crosshead.

The specimens were prepared in a laboratory. The concrete mix design used in this study is summarized in Table 2. The maximum gravel size (*G*_max_) was controlled to be 10 mm, the ratio of sand to aggregate (*S*/*a*) was fixed at 60% for all specimens. Ordinary Portland cement (OPC) was 480 to 482 kg/m^3^. An appropriate amount of water-reducing admixture (AD) was used to meet the target slump of 120 ± 25 mm, which was determined for tunnel shotcrete usage. The mixture was then mixed with PA fibers and placed in a mold for three days. After demolding, the specimens were cured at room temperature to represent PAFRC used in tunnel shotcrete. All specimens were tested on the 28th day. The 28-day compressive strength of the PAFRC was measured based on Ф100 × 200 mm cylindrical specimens. The slump and compressive strength of the specimens are presented in Table 3.

**Table 2:** Concrete mix proportions.

Specimen	*G*_max_ (mm)	*W*/*C* (%)	*S*/*a* (%)	Unit Weight (kg/m^3^)						WRA (C%)
				W	OPC	S	CS	G	Fiber	
PA7-RFA50 *	10	43.1	60	207	480	480	484	640	7.0	0.7
PA8-RFA50 *	10	43.1	60	207	480	480	484	640	8.0	0.8
PA9-RFA50 *	10	43.1	60	207	480	480	484	640	9.0	0.9
PA7-RFA60 *	10	43.8	60	211	482	381	576	635	7.0	0.8
PA8-RFA60 *	10	43.8	60	211	482	381	576	635	8.0	0.9
PA9-RFA60 *	10	43.8	60	211	482	381	576	635	9.0	1.0
PA10-RFA60 *	10	43.8	60	211	482	381	576	635	10.0	1.1

* RFA xx: ratio of recycled fine aggregate to fine aggregate.

**Table 3:** PAFRC slump and compressive strength.

Specimen	Slump (mm)	28-day Compressive strength (MPa)			
		SP-1	SP-2	SP-3	Avg.
PA7-RFA50	120	51.0	49.8	50.5	50.4
PA8-RFA50	115	51.5	50.2	51.8	51.0
PA9-RFA50	120	52.8	51.5	53.1	52.5
PA7-RFA60	115	47.5	49.6	49.5	48.9
PA8-RFA60	110	50.4	51.1	48.0	49.8
PA9-RFA60	120	49.7	48.9	51.4	50.0
PA10-RFA60	110	49.1	52.0	47.5	49.5

## 3. Experimental Results

### 3.1. Pull-Out Test Results

**Figure 7:**
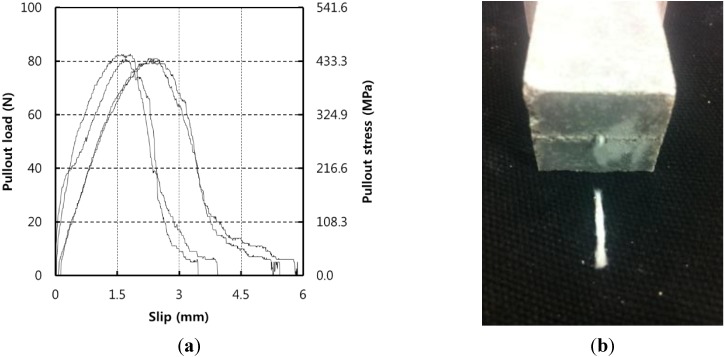
Pull-out test results. (**a**) Load-slip curve; (**b**) Fiber fracture.

Pull-out test results are presented in Figure 7. The PA fiber strength increased almost linearly as the displacement increased before peak loading. After peak loading, the PA fiber that was exposed outside the cement matrix started to untangle, and elongated. At this point, the micro PA fiber began to fracture. Contrary to other organic reinforcing fibers and steel fibers, the macro-size PA fiber broke without separating from the cement matrix. Steel fiber pull-out tests in other research [22] reported that the steel fiber was pulled out from the matrix at increasing loads. However, the macro PA fiber was not pulled out and instead fractured at increasing loads. Thus, the pull-out stress was computed to be for fiber fractured:

(1) $${\text{σ}}_{\text{min}}=\frac{4P_{\text{max}}}{\text{π}{d_{\text{f}}}^{2}}$$

where, σ_min_ is the minimum fiber-to-matrix bonding strength, which is greater than or equal to the PA fiber tensile strength; *P*_max_ is the maximum pull-out load; and *d*_f_ is the diameter of the PA fiber.

Generally, when the fiber is pulled out, such as the steel fiber during the pull-out test, the pull-out stress is computed as:

(2) $${\text{τ}}_{\text{max}}=\frac{P_{\text{max}}}{\text{π}d_{\text{f}}L_{\text{embed}}}$$

where, τ_max_ is the maximum fiber-to-matrix bonding strength based on the surface shear around the fiber; *L*_embed_ is the fiber embedment length in the matrix when debonding starts to initiate.

As shown in Figure 7, this PA pull-out phenomenon originates with the improved bonding strength of PA fiber attachment to the cement matrix. Because the macro PA fiber is made of 384 micro PA filaments and the specific surface area is significantly increased, the bonding strength between the macro PA fiber and cement matrix is greatly increased. Although the PA fiber was failed within the fibers, the fiber-to-matrix bonding strength was expected to be larger than or equal to the PA fiber tensile strength. Thus, Figure 7a was derived using Equation (1).

### 3.2. Flexural Bending Test Results

**Figure 8:**
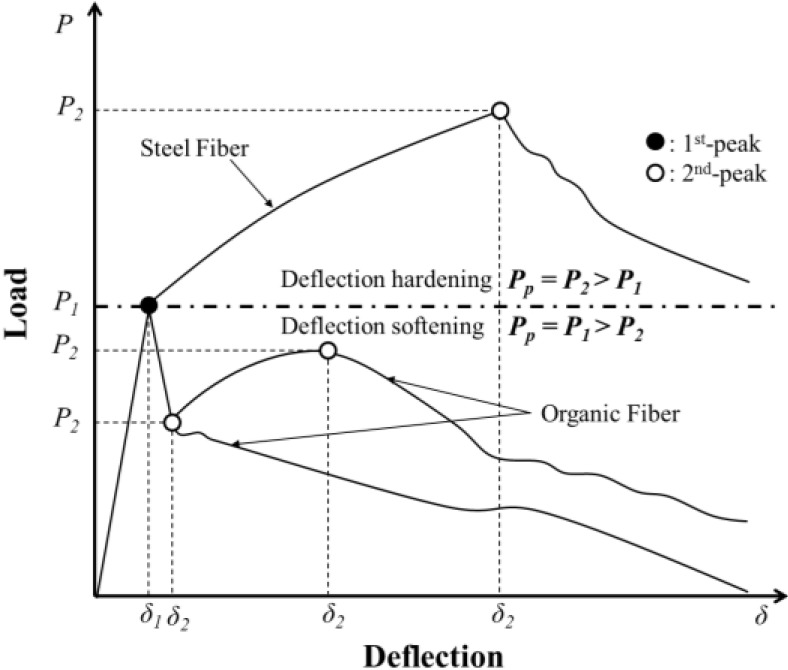
Typical load-deflection response of FRC.

The flexural responses of PAFRCs with varying fiber volumes of 7, 8, 9 and 10 kg/m3 were tested in accordance with ASTM C1609/C 1609M-05 [20]. As in Figure 8, this study identified two peak loads: a (1) first-peak load (*P*_1_) on the load-deflection, caused by the initiation of cracks in the specimen, and (2) second-peak load (*P*_2_) on the load-deflection when the PA fibers reached their ultimate strength. Corresponding strengths and deflections were denoted as *f*_1_ and δ_1_ for *P*_1_ and *f*_2_ and δ_2_ for *P*_2_, respectively. Residual loads ($P_{600}^{D}$ and $P_{150}^{D}$) and strength ($f_{600}^{D}$ and $P_{150}^{D}$) at a net deflection of *L*/600 and *L*/150 were measured for a beam with a depth of D. Toughness ($T_{150}^{D}$) and equivalent flexural strength ratio ($R_{T,150}^{D}$) at a net deflection of *L*/150 were also identified as follows:

(3) $$f_{1\;\text{or}\;2}=\frac{P_{1\;\text{or}\;2}L}{bd^{2}}$$

(4) $$R_{T,150}^{D}=\frac{150\cdot T_{150}^{D}}{f_{1}\cdot b\cdot d^{2}}\times 100\;(\%)$$

Based on the deflection (δ_1_) at the first-peak load, three additional points were investigated at 3δ_1_, 5.5δ_1_ and 10.5δ_1_ as per ASTM C1018 [19]. Current ASTM standard C1609 specifies δ_1_, δ_p_ (deflection at the peak load regardless of the first-peak or second-peak), *L/*600 and *L/*150. However, PAFRC occasionally exhibits a second peak that is larger than the first peak depending on the PA fiber volume content. This study intended to clearly identify whether pre- or post-cracking strength was larger. Also, PAFRC yields after cracking before the PA fiber reaches its ultimate strength, similar to hyper-elastic materials. Thus, additional points at 3δ_1_, 5.5δ_1_ and 10.5δ_1_ were used.

#### 3.2.1. Bending and Equivalent Bending Strength

**Figure 9:**
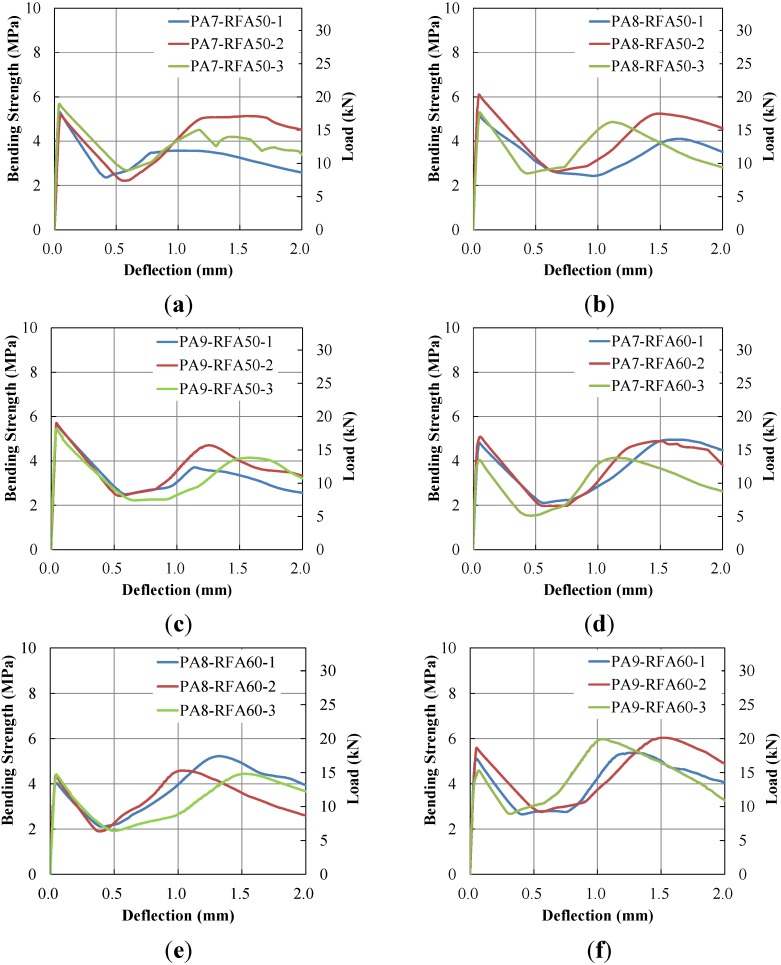

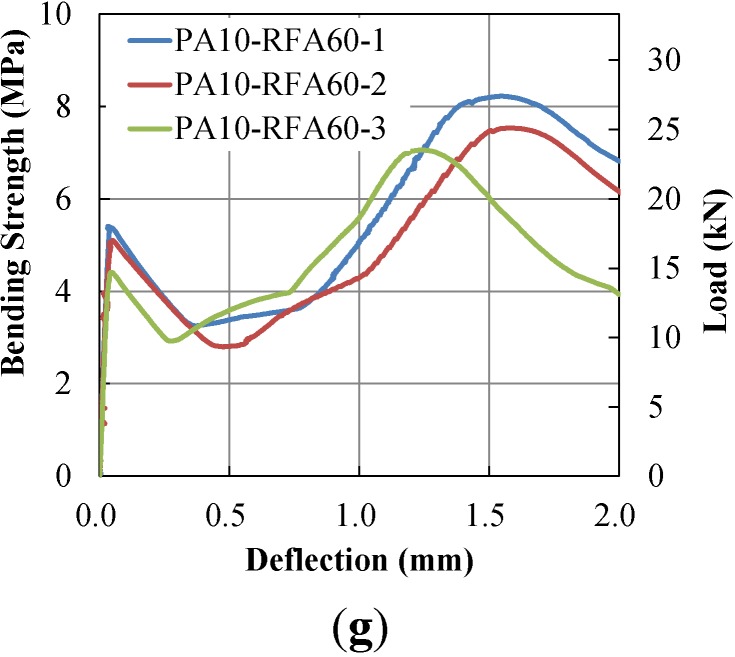
Load-deflection curves. (**a**) PA-7-RFA50; (**b**) PA-8-RFA50; (**c**) PA-9-RFA50; (**d**) PA-7-RFA60; (**e**) PA-8-RFA60; (**f**) PA-9-RFA60; (**g**) PA-10-RFA60.

Flexural bending tests of three specimens for each fiber volume content were conducted, as shown in Table 4. The bending strength (*f*_r_) and equivalent bending strength (*f*_e_) of each specimen was calculated based on the load-deflection curves in the following section (Figure 9), using the following equations:

(5) $$f_{\text{r}}=\frac{PL}{bd^{2}}$$

(6) $$f_{\text{e}}=\frac{A_{\text{b}}L}{{\text{δ}}_{150}bd^{2}}$$

where, *P* = peak load at δ_1_; *L* = span length (=300 mm); *b* and *h* = beam cross-section width and depth at the fracture surface, respectively; *A*_b_ = area under the load-deflection curves up to δ_150_ (N·mm) and δ_150_ = deflection of *L/*150 (=2.0 mm). The equivalent bending strength (*f*_e_) is derived from the equivalent flexural strength ratio, $R_{T,150}^{D}$, in Equation (4), which is equal to (*f_r_*/*f_e_*) × 100%.

For both RFA50 and RFA60, it is clearly observed that PA fiber improved the beam flexural strength, as in Table 4. Both bending strength and equivalent bending strength increased as fiber content increased from 7 to 10 kg/m^3^. However, the equivalent bending strength of PA9-FRA50 was smaller than PA7-RFA50 and PA8-RFA50. This phenomenon often occurs in other FRCs when the volume content of the reinforcing fiber is larger than a certain limit. Bending strengths of the RFA60 series were more significantly improved as PA fiber content was increased, compared to the RFA50 series. The RFA50 series produced larger bending strengths for PA7 and PA8, but the RFA60 series produced larger bending strengths for PA9 and PA10. To confirm that an excessive amount of PA fiber may drop the bending strength, similar to the RFA50 series, the PA10-RFA60 specimen was tested, but the bending strength continuously and significantly increased.

**Table 4:** Bending and equivalent bending strength.

Specimen	Bending strength (MPa)				Equivalent bending strength (MPa)			
	SP1	SP2	SP3	Average	SP1	SP2	SP3	Average
PA7-RFA50	5.31	5.18	5.69	5.39	3.28	4.11	3.86	3.75
PA8-RFA50	5.21	6.11	5.30	5.54	3.42	4.20	3.68	3.77
PA9-RFA50	5.59	5.71	5.46	5.59	3.32	3.68	3.34	3.45
PA7-RFA60	4.82	5.09	4.05	4.65	3.63	3.72	3.01	3.45
PA8-RFA60	4.04	4.39	4.42	4.28	3.77	3.36	3.24	3.46
PA9-RFA60	5.11	5.60	4.59	5.10	4.07	4.47	4.31	4.29
PA10-RFA60	4.51	5.10	4.41	4.97	5.60	5.09	4.78	5.16

#### 3.2.2. Load-Deflection Relationship of PAFRC

Load-deflection curves of three specimens were generated, as presented in Figure 9. Overall load-deflection curves were similar to polypropylene FRC [24,25,26,27,28,29] and synthetic FRC [30,31,32]. The load-deflection curves linearly increased up to the first-peak. After the first-peak, the loads were dropped approximately 50%. Then the curves exhibited convex shapes increasing and decreasing after the second-peak.

The PA fibers did not notably change the first-peak, but the post-cracking behaviors of all specimens were significantly improved. All specimens produced two peak bending strengths. The first-peaks apparently occurred near 0.005 mm of deflection and decreased up to 0.5 mm of deflection, which was approximately the lowest bending strength after the first-peaks. The load-deflection curves increased up to second-peaks until the deflections reached between 1.0 and 1.5 mm after the lowest peaks. The curves moderately decreased after the second-peaks, compared to the lines after the first-peaks.

The first-peak loads were 17975, 18468 and 18624 N for the RFA50 series and 15514, 14280, 16993 and 16572 N for the RFA60 series, respectively. The results show that the first-peak loads tended to increase as PA fiber content increased. However, the first-peak loads for PA8-RFA60 and PA10-RFA60 were smaller than for PA7-RFA60 and PA9-RFA60, respectively, because the first-peak loads are related to the concrete strength. Also, it should be noted that the first-peaks of the RFA50 series were larger than those of the RFA60 series, which was contrary to the bending strength, although the second-peaks of the RFA60 series were larger than those of the RFA50 series.

Initial PA fiber elongation was observed after the first-peak load. Since PA fiber tends to exhibit hyper-elasticity, a deflection occurs up to the inflection point and thereafter the load-deflection curve rapidly increases. In Figure 9, all PAFRC specimens exhibit the kinked (inflection) points after the first-peak near the *L/*600 and PA fiber elongation.

The second-peaks of the RFA50 series specimens were similar to the first-peaks. However, as discussed in Section 4.1, the second-peaks of the RFA60 series specimens exceeded the first-peaks. The ratios of the first to second-peak loads were 81.7, 85.6 and 74.5% for the RFA50 series and 100.2, 116.5, 113.8 and 153.0% for the RFA60 series. Detailed discussion regarding the flexural responses is provided in the following section.

## 4. Analysis

### 4.1. Flexural Responses of PAFRC

**Figure 10:**
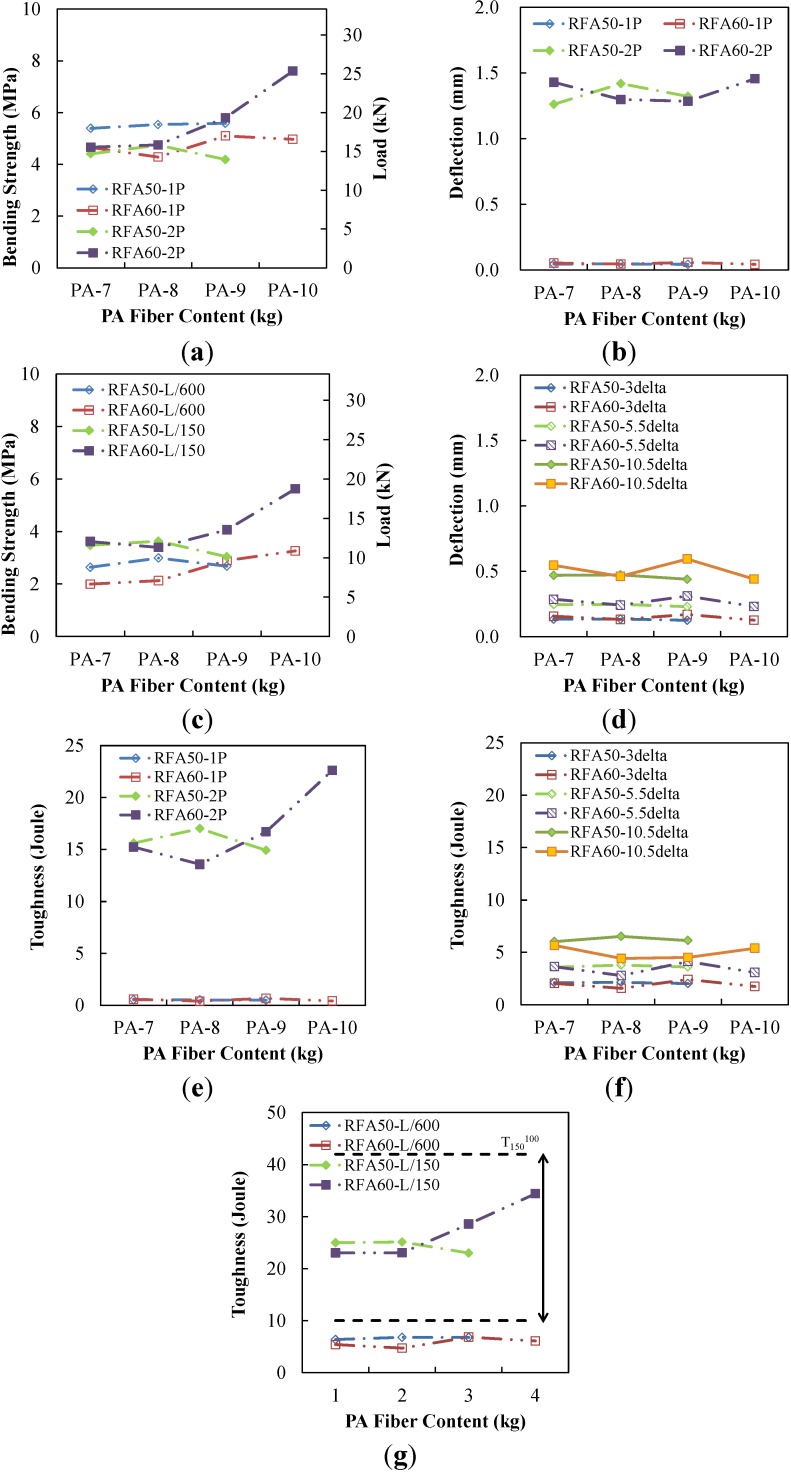
Average flexural responses of PAFRC. (**a**) Strength for first- and second-peak; (**b**) deflection for first- and second- peak; (**c**) strength for *L/*600 and *L/*150; (**d**) deflection for 3δ_1_, 5.5δ_1_ and 10.5δ_1_; (**e**) toughness for first- and second-peak; (**f**) toughness for 3δ_1_, 5.5δ_1_ and 10.5δ_1_; (**g**) PA-10-RFA60.

The flexural responses of PAFRC at δ_1_, δ_2_, 3δ_1_, 5.5δ_1_, 10.5δ_1_, *L/*600 and *L/*150 were investigated for loads, bending strength and toughness. The results are tabulated in Table 5 in the order of increasing deflections. The flexural responses in terms of bending strength, deflection and toughness are presented in Table 5 and Figure 10. Bending strength decreased from *f*_1_ to *f*_600_, and then increased up to *f*_2_ for all specimens. These bending strength fluctuations correspond to the load-deflection curves in Figure 9.

PA fiber content significantly influenced the second-peaks, as seen in Figure 10a,c. This results implies that the first-peaks are mainly dependent on the concrete strength. However, the deflections at the first-peaks were insignificantly influenced by PA fiber content as in Figure 10b,d. Toughness after the second-peak was significantly influenced by PA fiber content as seen in Figure 10e,f,g), while toughness before the second-peak was not. The PA fiber produces two peaks, as in Figure 9. Up to *L/*600 (=0.5 mm), which is an approximate lowest point, deflections are smaller than the second-peak and PA fiber bending strength is not exerted. However, PA fiber significantly improves the bending strength beyond *L/*600. Also, the toughness results of both the RFA 50 and 60 series at *L/*150 (=2.0 mm) lay between the maximum and minimum toughness ($T_{150}^{100}$) suggested by ASTM C1609/C1609M-10 [20].

**Table 5:** Average value of flexural responses of PAFRC.

Location	Response	PA-7-RFA50	PA-8-RFA50	PA-9-RFA50	PA-7-RFA60	PA-8-RFA60	PA-9-RFA60	PA-10-RFA60
First-peak	*P* (N)	17975	18468	18624	15514	14280	16993	16572
	δ (mm)	0.045	0.045	0.042	0.052	0.044	0.057	0.042
	*f* (MPa)	5.39	5.54	5.59	4.65	4.28	5.10	4.97
	*T* (Joule)	0.520	0.508	0.481	0.580	0.396	0.659	0.415
3δ_1_	*P* (N)	15678	16403	16538	13204	12087	14096	14653
	δ (mm)	0.134	0.135	0.125	0.156	0.132	0.170	0.126
	*f* (MPa)	4.70	4.92	4.96	3.96	3.63	4.23	4.40
	*T* (Joule)	2.110	2.135	2.019	2.039	1.567	2.408	1.751
5.5δ_1_	*P* (N)	13336	14095	14397	10257	9498	11402	12351
	δ (mm)	0.246	0.248	0.230	0.286	0.241	0.311	0.231
	*f* (MPa)	4.00	4.23	4.32	3.08	2.85	3.42	3.71
	*T* (Joule)	3.594	3.780	3.608	3.634	2.785	4.138	3.081
10.5δ_1_	*P* (N)	8681	10530	10213	6405	6686	10263	10781
	δ (mm)	0.469	0.473	0.439	0.546	0.461	0.594	0.441
	*f* (MPa)	2.70	3.16	3.06	1.92	2.01	3.08	3.23
	*T* (Joule)	6.037	6.530	6.135	5.666	4.420	4.518	5.395
*L/*600 (=0.50 mm)	$P_{600}^{100}$ (N)	8799	9972	8933	6647	7094	9658	10863
	$f_{600}^{100}$ (MPa)	2.64	2.99	2.68	1.99	2.13	2.90	3.26
	*T*_600_(Joule)	6.369	6.782	6.776	5.393	4.699	6.833	6.089
Second-peak	*P*_2_ (N)	14685	15807	13954	15540	15843	19333	25357
	δ_2_ (mm)	1.261	1.419	1.321	1.429	1.298	1.284	1.456
	*f*_2_ (MPa)	4.41	4.74	4.19	4.66	4.75	5.80	7.61
	*T*_2_ (Joule)	15.596	17.003	14.902	15.225	13.568	16.695	22.616
*L/*150 (=2.00 mm)	$P_{150}^{100}$ (N)	11589	12107	10129	12059	11320	13554	18755
	$f_{150}^{100}$ (MPa)	3.48	3.63	3.04	3.62	3.40	4.07	5.63
	*T*_150_ (Joule)	24.981	25.114	22.982	23.019	23.046	28.569	34.401
	$R_{T,150}^{100}$(%)	69.6	67.9	61.7	74.2	81	84.5	104.0

### 4.2. Toughness Performance Levels

The toughness performance levels (TPL) by Chen (1995) [32] were used to specify the PAFRC toughness information. As discussed in the last paragraph of Section 4.1, the PAFRC toughness lay between the maximum and minimum of ASTM C1609/C1609M-10 [20]. However, this information is not enough to explain the toughness characteristics of PAFRC and therefore, a detailed description is required to verify compliance with construction specifications, or quality control, of in-service PAFRC. In tunneling practice, the TPL by Chen [33] (1995) is commonly used for steel fiber reinforced shotcrete.

**Figure 11:**
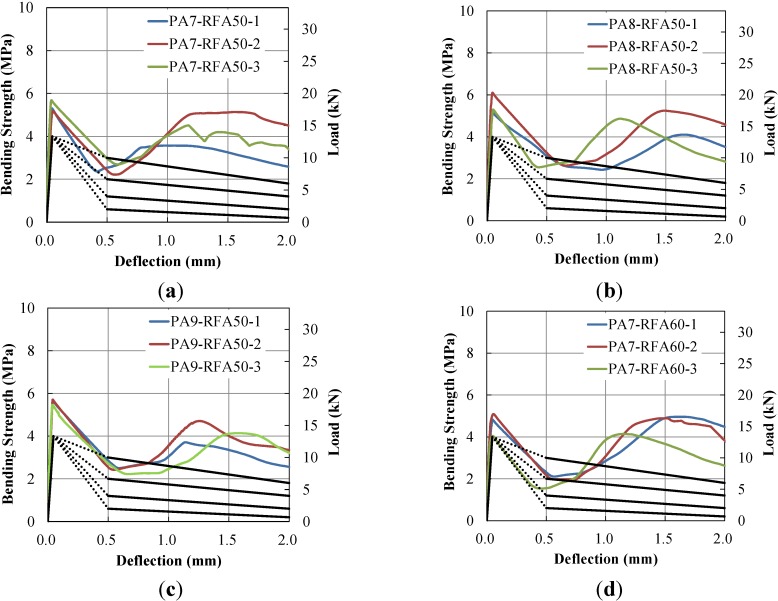

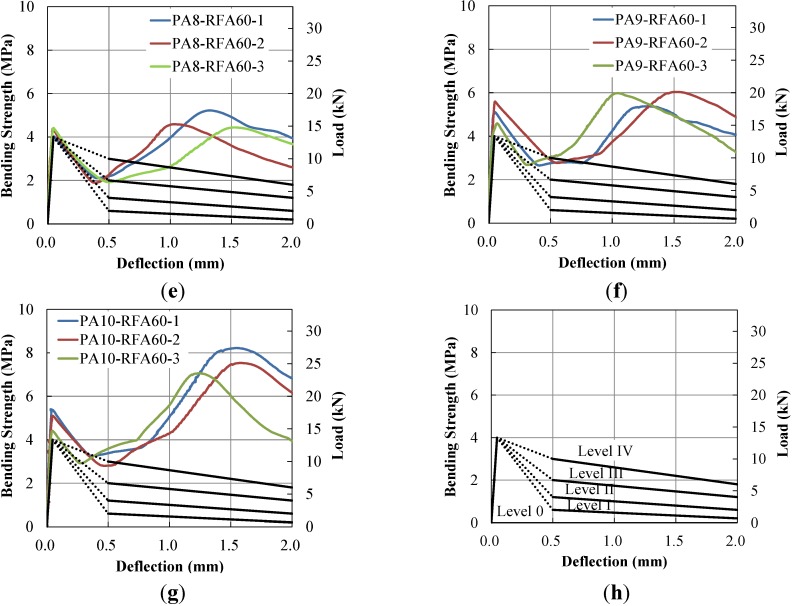
Toughness performance level. (**a**) PA-7-RFA50; (**b**) PA-8-RFA50; (**c**) PA-9-RFA50; (**d**) PA-7-RFA60; (**e**) PA-8-RFA60; (**f**) PA-9-RFA60; (**g**) PA-10-RFA60; (**h**) TPL Levels.

The TPL is based on nominal residual flexural strengths based on the load-deflection curves at δ_1_, *L/*600 and *L/*150. The TPL defines five different toughness levels and corresponding residual loads, as in Table 6. Equivalent flexural toughness parameters to TPL levels can be found in Chen (1995). The residual loads here were computed based on the 100 × 100 × 300 mm specimen size. A design flexural strength of 4.0 MPa, which is equivalent to a design flexural load of 13,333 N, was adopted in this study as it is a common practice in tunnel shotcrete. It should be noted that the TPL does not consider the unstable region in the load-deflection curves, *i.e.* from the first-crack (δ_1_) to *L/*600 (=0.5 mm). Load-deflection curves of each specimen were investigated to determine the TPL levels presented in Figure 11.

**Table 6:** Toughness performance level criteria.

TPL Level	Residual loads (% of Design flexural strength)		
	at δ_1_ (=0.047 mm)	at *L/*600 (=0.5 mm)	at *L/*150 (=2.0 mm)
0	No fiber reinforced		
I	13333 (100%)	2000 (15%)	667 (5%)
II	13333 (100%)	4000 (30%)	2000 (15%)
III	13333 (100%)	6667 (50%)	4000 (30%)
IV	13333 (100%)	10000 (15%)	6000 (45%)

The determined TPL levels are presented in Table 7. This study investigated at three points and took the lowest level among all specimens for each specimen type. It is clearly shown in Table 7 that all specimens of the RFA50 series satisfied Level III. However, for the RFA60 series, specimens with lower PA fiber content exhibited Level II, and those with higher PA fiber contents exhibited Level III. Therefore, the RFA60 series requires greater PA fiber content compared to the RFA50 series. Moreover, all the performance levels satisfied Level IV at δ_1_ and *L/*150 (=2.0 mm) although lower performance levels were found at *L/*600 (=0.5 mm). As seen in Figure 11, the load-deflection curves rapidly decreased after the first-peaks, but the loads increased as the deflection increased to satisfy Level IV. This finding implies that the TPL analysis by Chen (1995) is not enough to explain the full toughness characteristics of PAFRC that has a sudden drop after the first-peak in the load-deflection curve.

**Table 7:** Toughness performance levels.

Specimen	at δ_1_				at *L/*600 (=0.5 mm)				at *L/*150 (=2.0 mm)				TPL Level
	SP1	SP2	SP3	Lvl.	SP1	SP2	SP3	Lvl.	SP1	SP2	SP3	Lvl.	
PA7-RFA50	IV	IV	IV	IV	III	III	III	III	IV	IV	IV	IV	III
PA8-RFA50	IV	IV	IV	IV	IV	IV	III	III	IV	IV	IV	IV	III
PA9-RFA50	IV	IV	IV	IV	III	III	III	III	IV	IV	IV	IV	III
PA7-RFA60	IV	IV	IV	IV	III	III	II	II	IV	IV	IV	IV	II
PA8-RFA60	IV	IV	IV	IV	III	III	II	II	IV	IV	IV	IV	II
PA9-RFA60	IV	IV	IV	IV	III	III	IV	III	IV	IV	IV	IV	III
PA10-RFA60	IV	IV	IV	IV	IV	III	IV	III	IV	IV	IV	IV	III

## 5. Conclusions

This study described the development and processing of a macro-sized PA fiber to be used in concrete reinforcement, and then investigated the influence of the PA fiber on flexural responses in accordance with ASTM standards C1018-97 and C1609/C 1609M-05. The conclusions derived from this study are as follows:

- The compressive strength and the first-peak were not notably influenced by the PA fiber contents because PA fiber contents considered in this study did not deteriorate the compressive strength and PA fibers were intended to resist tensile stresses. However, the PA fiber significantly improved flexural responses and toughness after the deflection of *L/*600 as the PA fiber content was increased. Before *L/*600 deflection, PA fiber tensile strength was so low that allowed the first crack of concrete specimens, but after *L/*600 deflection, PA fibers started to exert high tensile strength and increased ductility and toughness of concrete.
- The PA fibers produced two peak bending strengths. The first-peaks apparently occurred near 0.005 mm of deflection and decreased up to 0.5 mm (*L/*600) of deflection, which was the approximately lowest bending strength after the first peak. Then the bending strength increased up to second peak until the deflections reached between 1.0 and 1.5 mm after the lowest peaks. The load-deflection curve decreased moderately after the second-peak compared to the decreasing slope after the first-peak.
- Toughness performance level (TPL) results exhibited Level III for all specimens in the RFA50 series based on the design flexural strength of 4.0 MPa. However, for the RFA60 series, specimens with less than 8 kg of PA fiber content were determined to be Level II, and specimens with 9 and 10 kg of PA fiber content were determined to be Level III. Therefore, the RFA60 series requires greater PA fiber content compared to the RFA50 series. However, due to the PA fiber characteristic, load-deflection curves exhibited that PAFRC satisfy Level IV at δ_1_ and *L/*150 (=2.0 mm) except at *L/*600 (=0.5 mm). Therefore, the TPL analysis is not enough to explain the full toughness characteristics of PAFRC that has a drop after the first-peak in the load-deflection curve.
